# Supplementary material for: Schizosaccharomyces pombe Telomerase RNA: Secondary Structure and Flexible-Scaffold Function
Source: Mol Cell Biol. Author manuscript; Available in PMC 2026 Apr 8. (PMC13058752; doi:10.1080/10985549.2025.2571189)
Supplement: Supp 1 [file NIHMS2120146-supplement-Supp_1.pdf]

Supp. Fig. 1

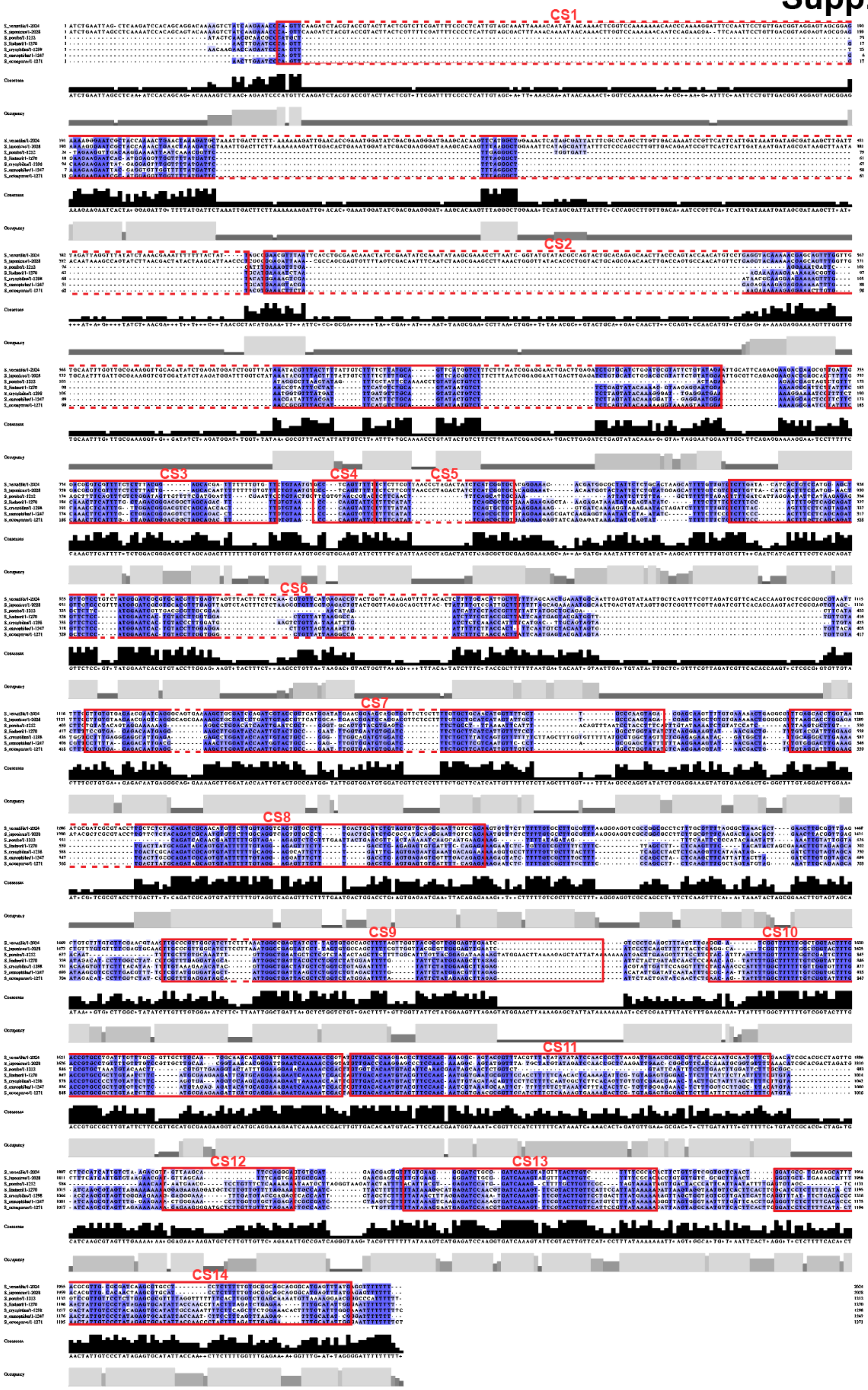

Supplementary Figure 1. Alignment of TER1 telomerase RNA gene from 7 *Schizosaccharomyces* species. *MUSCLE* was used to generate a multiple-sequence alignment of TER1 from the 7 species. The alignment was visualized using *Jalview* software and exported with base identities highlighted. Darkest blue color indicates 100% conserved nucleotides, with lighter tones indicating somewhat lower levels. Red boxes indicate the conserved sequence (CS) regions 1–14, which are also indicated on the TER1 secondary structure in Figure 1. A Jalview-generated consensus sequence is indicated below the aligned sequences.

Supp. Fig. 2

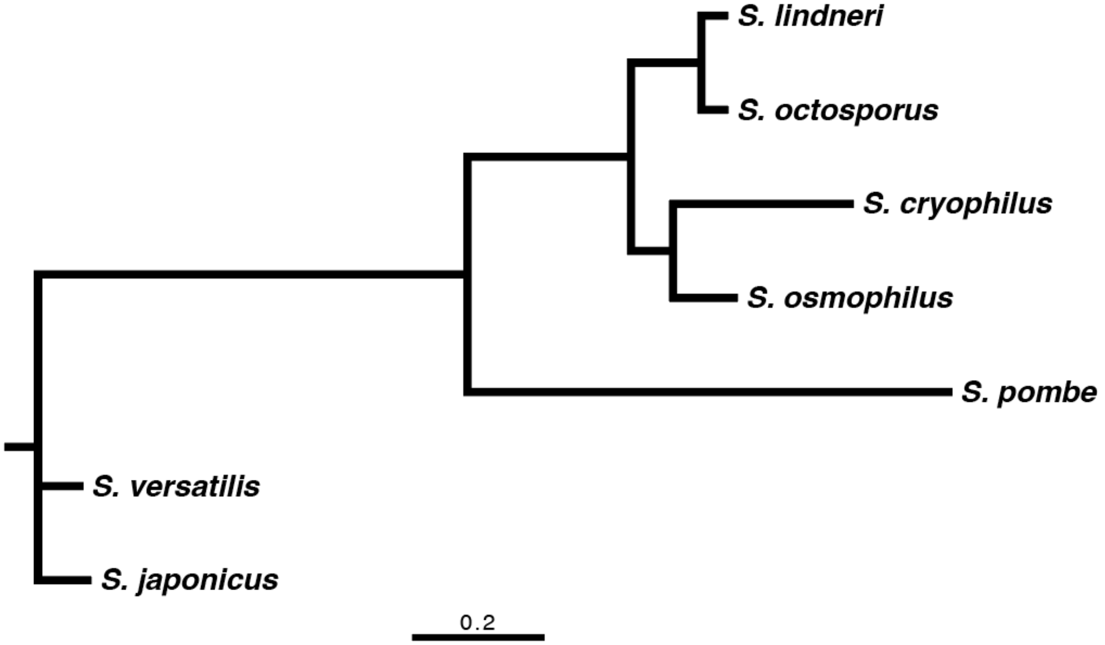

Supplementary Figure 2. Phylogenetic tree showing relatedness of the 7 *Schizosaccharomyces* species' TER1 genes. *IQTree* was used to generate the phylogenetic tree for TER1 based on the alignment in Figure 1, which was then visualized using *FigTree* software. The sequences of 5 species (*S. lindneri*, *octosporus*, *cryophilus*, *osmophilus*, and *pombe*) are more similar to each other than the outgroup containing *S. versatilis* and *japonicus*. Scale bar, rates of change based on *IQTree* calculations.

Supp. Fig. 3

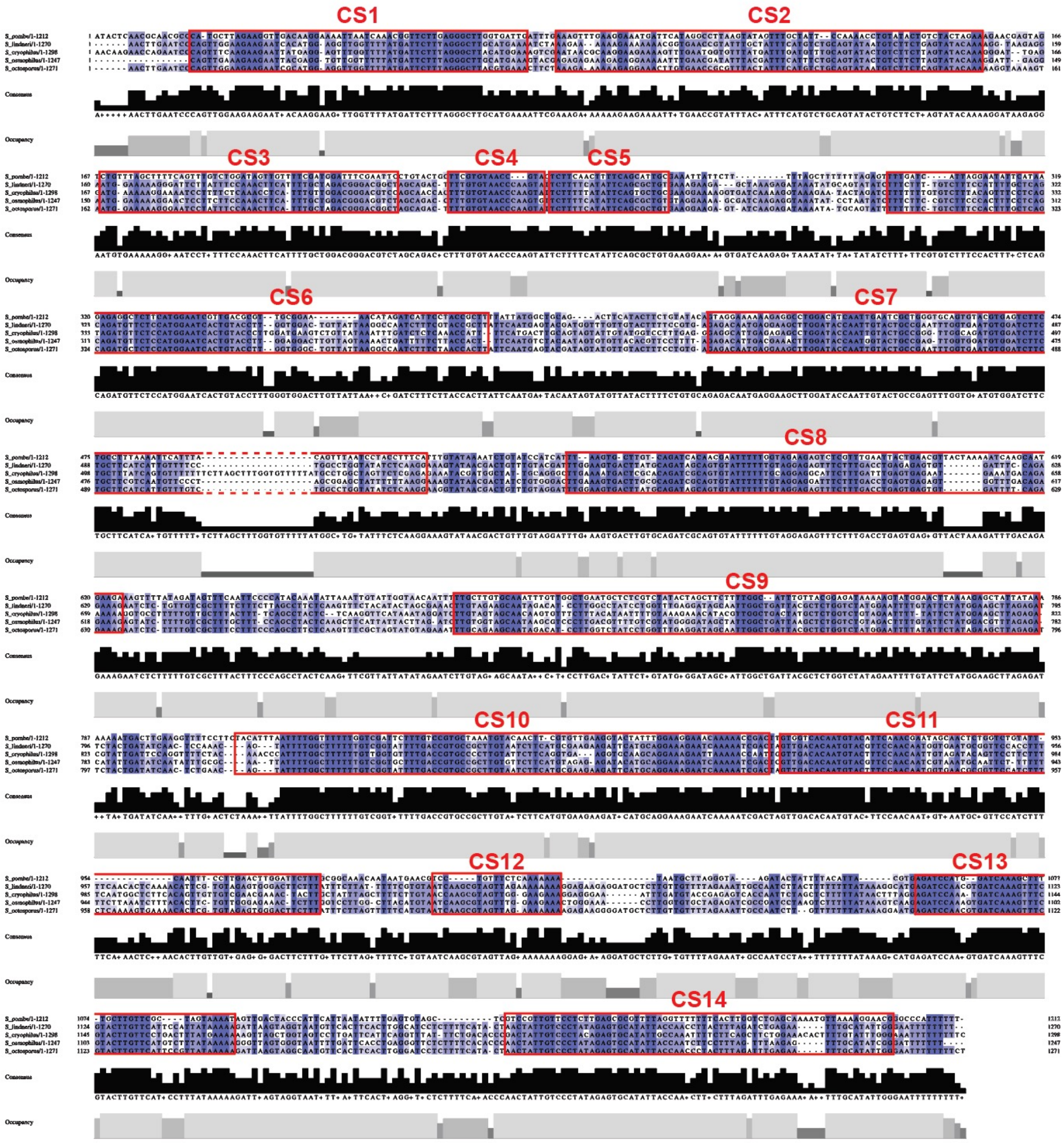

Supplementary Figure 3. Alignment of TER1 from the *Schizosaccharomyces* species most related to *S. pombe*. As in Supplementary Figure 1, TER1 sequences were aligned from 4 species most related to *S. pombe*, and thus 5 are shown in the alignment output, as visualized using *Jalview* software. Darker blue color indicates greatest conservation, whereas white regions are not conserved in sequence. Red boxes indicate the conserved sequence (CS) regions as shown in Figure 1 (secondary structure model of TER1) and Supplementary Figure 1 (7-species alignment). A Jalview-generated consensus sequence is indicated below the aligned sequences.

Supp. Fig. 4

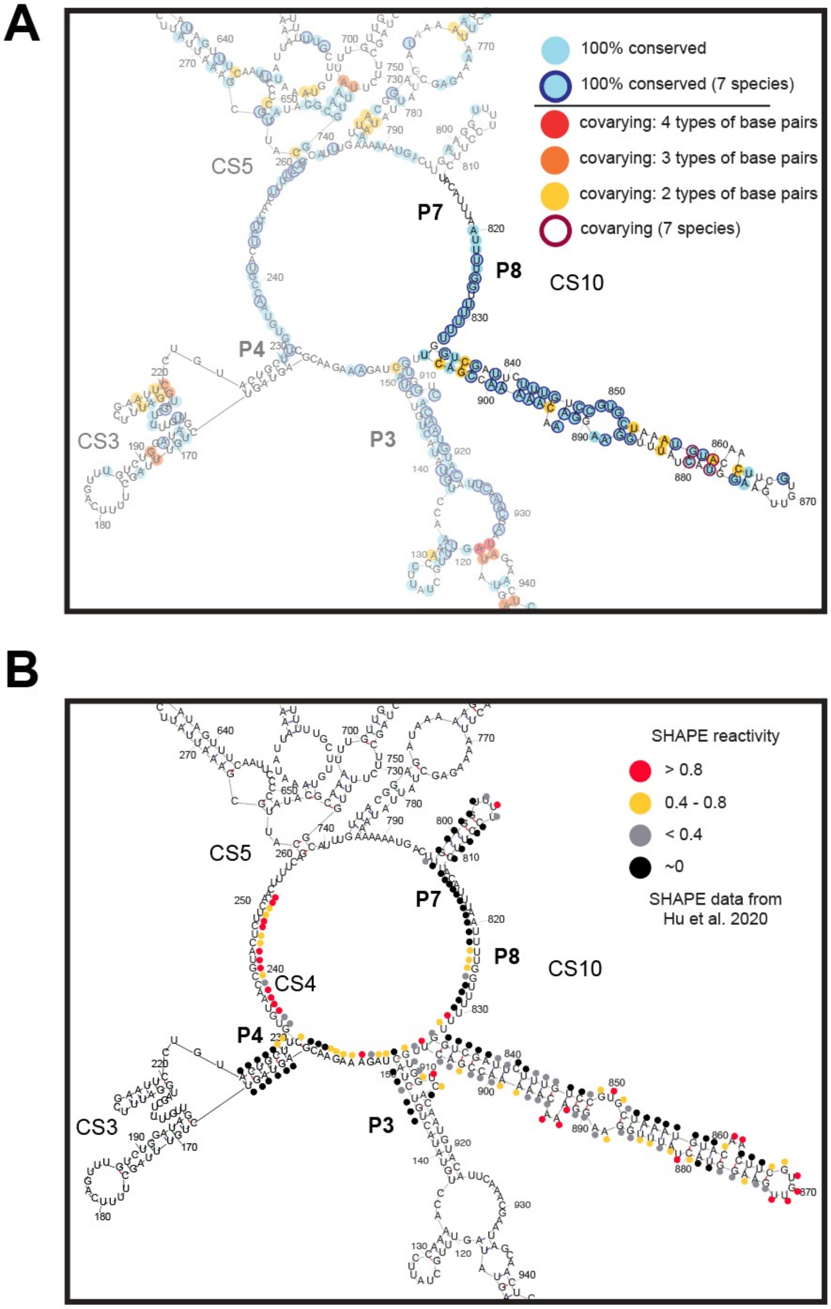

Supplementary Figure 4.

Proposed secondary structure model of the core of TER1 (without the pseudoknot or base triples). The model shown in both A and B was determined using *Mfold* (which does not predict pseudoknots) and forcing the base pairs listed in Supp. Table 1, which are supported by covarying nucleotides during evolution. The structural model is the same as Figure 1 (where the higher-order folded state including the reported pseudoknot is shown) except for the region that is not faded-out in **A**; i.e., the residues that give rise to the modeled pseudoknot.

A. Conservation and covariation data mapped onto our secondary structure model (i.e. without the pseudoknot). Some base pairs are separated in the pseudoknot but are predicted in the secondary structure model, which could be a folding intermediate (pseudoknots are higher-order RNA structures that fold after RNA initial base-pairing interactions in helices).

B. SHAPE reactivity data from Hu *et al.* 2020 mapped onto our secondary structure model of the core.

Supp. Fig. 5

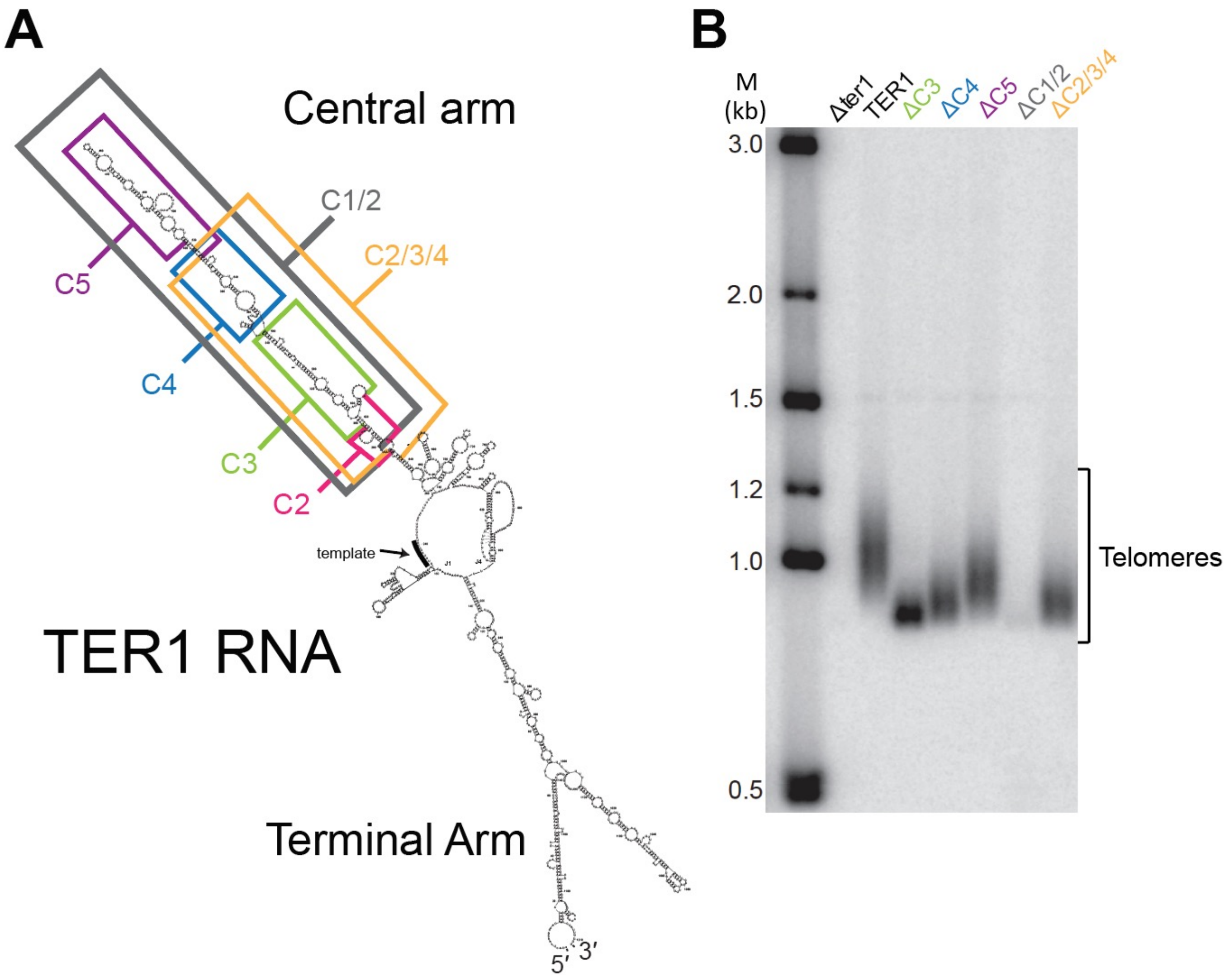

Supplementary Figure 5.

Telomere maintenance in *S. pombe* cells with TER1 Central-Arm truncations.

A. Schematic of Central-Arm truncations cloned and tested for complementation *in vivo*. Boxes indicate the sections of the Central Arm removed and expressed from a plasmid in *S. pombe ter1*Δ cells.

B. Truncations tested in the Central Arm lead to shorter telomeres. Telomere Southern blot showing telomeres of the indicated alleles after passaging cells.

Supp. Fig. 6

A

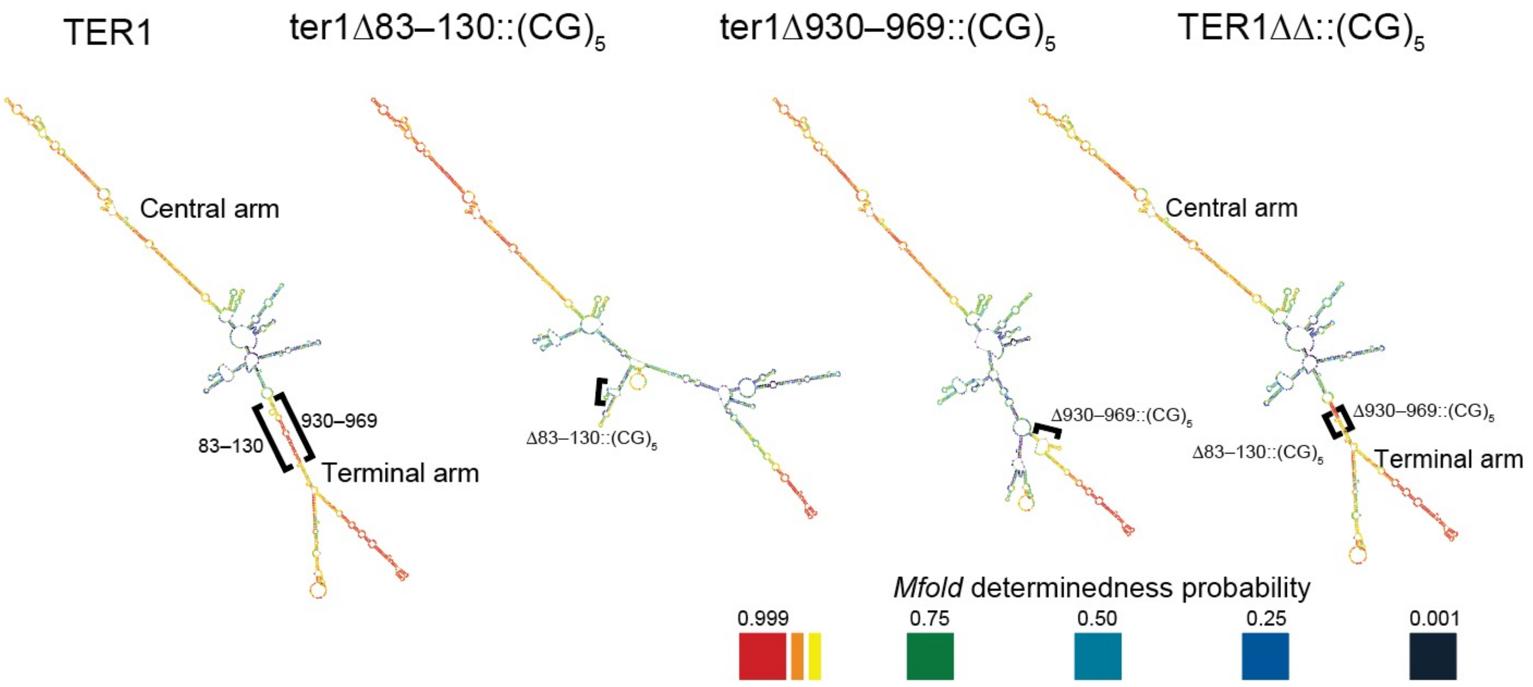

B

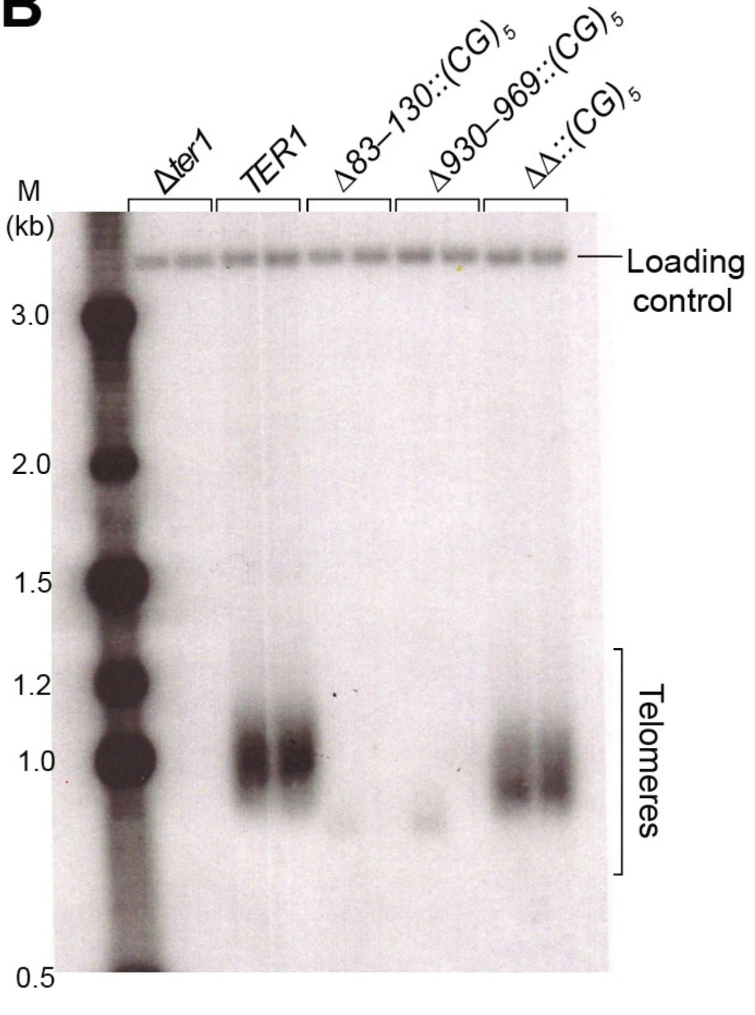

Supplementary Figure 6.

Experimental evidence for the Terminal Arm: compensatory base-substitution mutants on each side of the predicted arm allow function *in vivo* when combined.

A. The nucleotides substituted with $(CG)_5$ on each side of the Terminal Arm are shown on the secondary structures. Since phylogenetic information (covarying base pairs in evolution) are not appropriate to use for modeling of *mutant* TER1 RNAs, bioinformatic lowest-free-energy modeling was employed to compare predicted folding of mutant RNAs compared to wild type. Thus, the models shown in **A** were determined using solely *Mfold*. (Note: this software does not predict pseudoknots.) The rainbow color spectrum in the folds shown is from the output of the *p-num* (or *SIR_GRAPH*) feature of *Mfold* (Zuker and Jacobson, *RNA* 1998). The red end of the spectrum has energetically higher-confidence, or "determinedness," of the folding prediction based on *Mfold*'s lowest-free-energy calculation algorithm; i.e., the high-determinedness folded conformations also appear in slightly less-negative-ΔG calculations. Note that the overall folding of TER1 is predicted to be largely restored in the ΔΔ mutant compared to the single Δ alleles.

B. Telomeres are lost in single mutants, whereas they are restored in the double-mutant allele to near wild-type length, strongly suggesting these regions are paired in the wild-type TER1 telomerase RNA.

Supp. Fig. 7

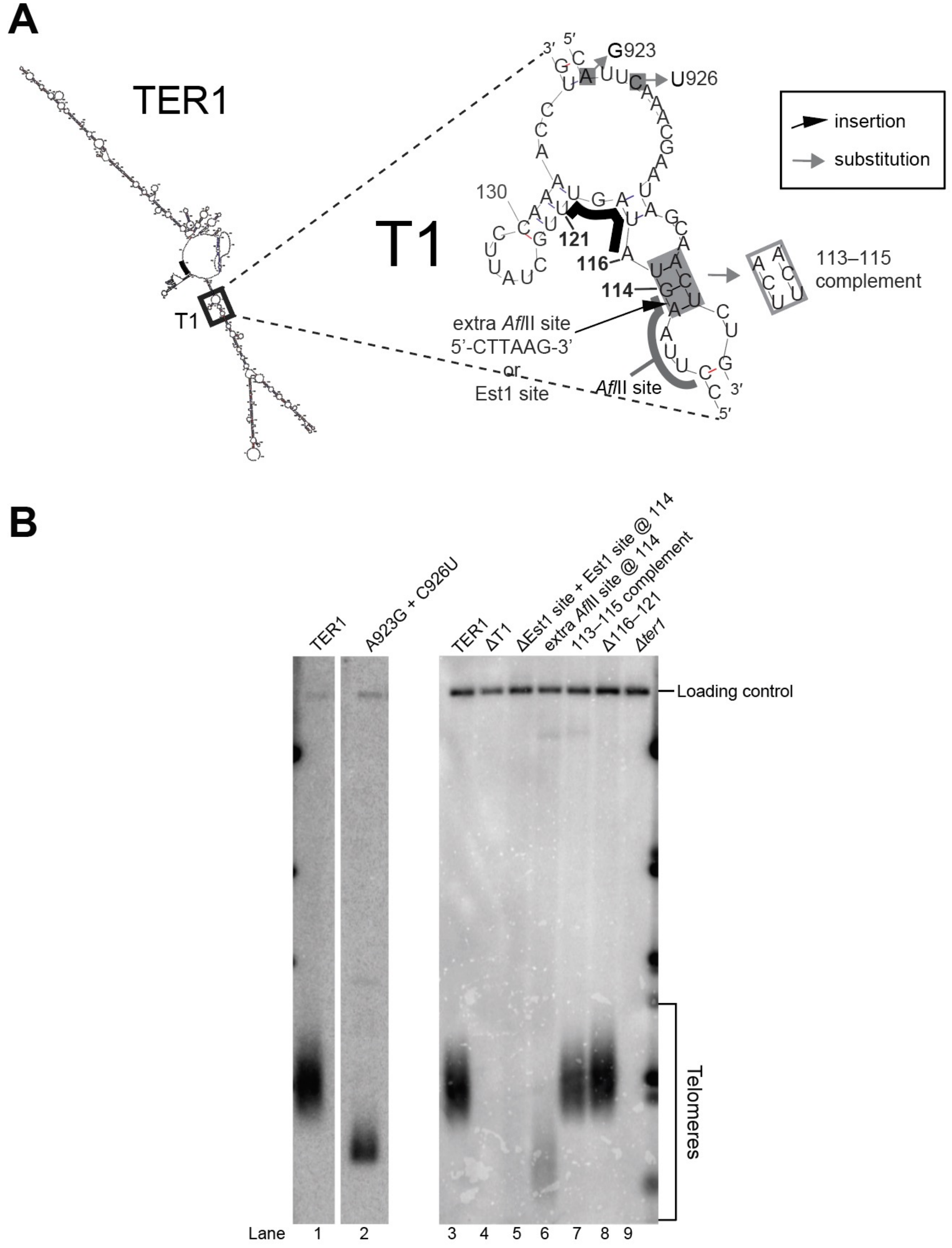

Supplementary Figure 7

Mutational analysis of the essential T1 region of TER1.

A. Schematic of the mutants generated in the T1 region. Mutants designed and tested *in vivo* include deletion (ΔT1), truncations (Δ116–121), nucleotide substitutions (A923G+C926U, insertions (extra *Afl*II site@114), and a predicted base-pair substitution (113–115 complement).

B. Telomere Southern blot of T1 element-dissecting mutants.

Supplementary Table 1

Base pairs used to constrain *Mfold model based on phylogenetic information*, etc.

Numbering is based on *S. pombe* TER1 RNA nts.

List of base pairs forced in *Mfold*. *RNAalifold* was used to determine likely-covarying base pairs. These, along with base pairing data from Box *et al.* 2008, were used to constrain the *Mfold* to determine the lowest free-energy structure that also includes all the base pairs listed. Numbering is based on the 1212-nt *S. pombe* TER1.

Primary sequence order

5' nt	3'nt
42	1165
51	1156
53	1154
55	1151
61	1145
69	997
73	993
91	977
92	976
117	935
118	934
150	235
153	232
163	229
164	228
165	227
166	226
172	191
208	220
209	219
211	217
260	737
264	651
296	623
327	578
330	575
340	561
341	560
366	553
376	544
377	543
390	534
391	533
402	518
407	513
408	512
415	507
419	487
426	480
427	479
428	478
436	469
438	468
449	460
451	458
656	691
696	733
711	718
745	784
746	783
757	769
834	905
836	903
845	895
1003	1135
1043	1093
1047	1089
1050	1084
1070	1075

Rank order

5' nt	3' nt
118	934
711	718
117	935
208	220
407	513
172	191
408	512
92	976
69	997
426	480
757	769
341	560
73	993
209	219
391	533
211	217
330	575
340	561
377	543
419	487
53	1154
428	478
438	468
1050	1084
746	783
656	691
451	458
1003	1135
376	544
260	737
264	651
1047	1089
427	479
390	534
402	518
327	578
1043	1093
745	784
836	903
845	895
449	460
55	1151
696	733
51	1156
42	1165
415	507
1070	1075
834	905
436	469
366	553
61	1145
91	977
150	235
153	232
296	623
TBE bps from Box et al 2008:	
163	229
164	228
165	227
166	226

Supplementary Table 2

Allele	Plasmid	Nucleotides deleted (truncation alleles)	Total # of nts deleted (truncation alleles)
**WT Ter1**	pDZ995		
**Ter1 core (528)**	pDZ983		
**Micro-Ter1 (623)**	pDZ984		
**ΔC1**	pDZ516	298-612	315
**ΔC2**	pDZ517	276-297, 613-630	40
**ΔC1/2**	pDZ321	276-630	355
**ΔC3**	pDZ318	298-343, 558-612	101
**ΔC4**	pDZ319	345-396, 524-556	85
**ΔC5**	pDZ320	402-525	124
**ΔC2/3/4**	pDZ322	277-394, 524-637	232
**ΔT1**	pDZ510	108-134, 924-945	49
**ΔT2**	pDZ515	47-64, 1142-1206	83
**ΔT3**	pDZ514	988-1002, 1127-1141	30
**ΔT4**	pDZ513	1003-1035, 1096-1126	64
**ΔT5**	pDZ512	1036-1095	60
**ΔT6**	pDZ325		
**T5@348**	pDZ985		
**T5@185**	pDZ986		
**T6@348**	pDZ675		
**Rev comp T5@348**	pDZ987		
**WT + T5@348**	pDZ988		
**WT + T5@185**	pDZ989		
**83–130::(CG)5**		83-130	48
**930–969::(CG)5**		930-969	40
**83–130 + 930–969::(CG)5**		83-130, 930-969	88
**T1 113–115 complement**	pDZ991		
**T1 Δ116–121**	pDZ990	116-121	6
**Est1@109**	pDZ676		
**T1 point mutants 923 + 926**	pDZ992		
**T1 w/ extra *Afl* II site**	pDZ993		
**Vector**	pDZ994		
			Total dispensable nts: 557
